# Estudios Caso-Control: Posible Ahorro de Tiempo y de Recursos, y Posible Ganancia de Potencia Estadística

**DOI:** 10.31083/RN43204

**Published:** 2025-11-27

**Authors:** Carmen Carazo-Díaz, Luis Prieto-Valiente

**Affiliations:** ^1^Facultad de Medicina, Universidad Católica de Murcia (UCAM), 30107 Murcia, España; ^2^Sociedad Científica de Investigación Biomédica (SCIB), 07010 Palma de Mallorca, España

**Keywords:** caso-control, estudio prospectivo, riesgo relativo, odds ratio, epidemiología pública, case-control, prospective study, relative risk, odds ratio, public epidemiology

## Abstract

Aunque los estudios prospectivos estiman directamente la influencia de un factor en la aparición de una enfermedad, en muchas ocasiones los muestreos Caso-Control son más baratos, rápidos, y a veces los únicos factibles. En ellos se toma una muestra aleatoria de individuos enfermos (casos) y se evalúa la proporción de ellos, P_1_, que estaban expuestos al factor. También se toma una muestra aleatoria de no enfermos (controles) y se evalúa la proporción de ellos, P_0_, que estaban expuestos. A partir de estas dos proporciones se calculan las respectivas Odds y su cociente, la Odd Ratio, OR. Cuando la incidencia de la enfermedad es pequeña, digamos menor del 10%, este valor de la OR se aproxima mucho al Riesgo Relativo, RR, y por ello nos dice, con buena aproximación, cuánto mayor es el riesgo de tener la enfermedad si se está expuesto al factor. En todos los casos los estudios caso control suelen tener más factores de confusión, no fácilmente controlables, que los estudios prospectivos. Pero en algunas circunstancias tienen potencia estadística muy superior al diseño prospectivo.

## Abstract (en)

Although prospective studies directly estimate the influence of a factor on the onset of a disease, case-control sampling is often cheaper, faster, and sometimes the only feasible option. In these studies, a random sample of individuals with the disease (cases) is taken, and the proportion of them, P_1_, who were exposed to the factor is evaluated. A random sample of individuals without the disease (controls) is also taken, and the proportion of them, P_0_, who were exposed is evaluated. From these two proportions, the respective odds and their ratio, the odds ratio (OR), are calculated. When the incidence of the disease is small, say less than 10%, this OR value closely approximates the relative risk (RR), and therefore tells us, with good approximation, how much greater the risk of having the disease is if one is exposed to the factor. In all cases, case-control studies tend to have more confounding factors, which are not easily controllable, than prospective studies. However, under certain circumstances, they have a much higher statistical power than the prospective design.

## 1. Introducción

Uno de los principales objetivos de los estudios epidemiológicos es detectar asociaciones entre procesos patológicos y los llamados “Factores de Riesgo”, que son toda característica biológica o ambiental que pueda afectar a la incidencia de la patología, a su evolución o trasmisión. Puede haber también “Factores Preventivos” que afectarían beneficiosamente a la aparición o evolución de la enfermedad.

Tradicionalmente para ver la influencia de un factor en la aparición de una enfermedad se realizaban lo que se llaman Estudios Prospectivos, en los que se toman dos muestras aleatorias, una de individuos expuestos al factor y otra de no expuestos, y se calcula en cada una de ellas la proporción de enfermos. Pero otra opción son los estudios retrospectivos Caso-Control, que veremos a continuación [1].

## 2. Tipos de Muestreo: Prospectivo y Caso Control

Como en la práctica no se estudian poblaciones sino muestras de individuos tomadas de esas poblaciones, veamos qué cantidades poblacionales se pueden estimar dependiendo de cómo se haga el muestreo.

Sea la población con 300.000 habitantes, en la que 100.000 fuman y 40.000 de los fumadores y 10.000 de los no fumadores tienen la enfermedad D, ver Tabla 1.

**Tabla 1.** **Descripción de la población considerada como ejemplo, donde D+ son enfermos y D^–^ son sanos**.

	Fuman	No Fuman	TOTAL	
D +	A = 40.000	B = 10.000	M_1_ = 50.000	P_1_ = 4/5 = 0,80
D^–^	C = 60.000	D = 190.000	M_0_ = 250.000	P_0_ = 6/25 = 0,24
Tot	N_1_ = 100.000	N_0_ = 200.000	N = 300.000	P_1_/P_0_ = 0,80/0,24 = 3,33
	R_1_ = 0,40	R_0_ = 0,05	RR = 0,40/0,05 = 8,00	

Vemos que son enfermos 5% de los no fumadores y 40% de los fumadores, porcentajes que se pueden estimar haciendo muestreo prospectivo, que comentamos enseguida. Y vemos que son fumadores 24% de los no enfermos y 80% de los enfermos, porcentajes que se pueden estimar haciendo muestreo caso-control, que comentamos a continuación.

También vemos que la Odd Ratio, OR tiene el mismo valor calculada con las proporciones de enfermos que con las proporciones de fumadores [2]. En efecto:

OR de fumar calculada con las P_1_ y P_0_: (0,8/0,2)/(0,24/0,76) = 4/0,3158 = 12,67, es decir, el número de fumadores por cada no fumador es casi 13 veces mayor en enfermos que en sanos.

Y OR de enfermar calculada con las R_1_ y R_0_: (0,4/0,6)/(0,05/0,95) = 0,667/0,052 = 12,67, es decir, el número de enfermos por cada no enfermo es casi 13 veces mayor en fumadores que en no fumadores.

Muestreo prospectivo: Se toma una muestra aleatoria de fumadores y se cuenta la cantidad de ellos que desarrollan la enfermedad. La correspondiente frecuencia relativa muestral estima el R_1_ poblacional. También se toma una muestra aleatoria de individuos no fumadores y se cuenta la cantidad de ellos que desarrollan la enfermedad. Esta frecuencia relativa muestral estima el R_0_ poblacional. El cociente de estos dos estimadores estima el Riesgo Relativo, RR poblacional. Con este muestreo no se puede estimar P_1_ ni P_0_ ni el cociente P_1_ /P_0_ [3]. Estas tres cantidades suelen ser de menor interés para médicos y pacientes.

Muestreo caso-control: Se toma una muestra aleatoria de individuos enfermos y se evalúa la frecuencia relativa de ellos que estaban expuestos, es decir, que fumaban. Esta frecuencia relativa muestral estima la correspondiente P_1_ poblacional. También se toma una muestra aleatoria de no enfermos y se evalúa la frecuencia relativa de ellos que estaban expuestos. Esta frecuencia relativa muestral estima la P_0_ poblacional [3, 4]. Con este muestreo no se puede estimar R_1_ ni R_0_ ni el cociente R_1_/R_0_ = RR, que suelen ser de interés para médicos y pacientes, porque los valores muestrales que estimarían R_1_ y R_0_ dependen de la cantidad de Casos y Controles que se haya decidido tomar.

Veamos cómo varían los “presuntos estimadores” de R_1_ y R_0_ de la anterior población en dos muestras Caso-Control concretas, A y B.

A - Muestreo Caso-Control tomando una muestra aleatoria de 200 enfermos y otra muestra aleatoria de 25 sanos y asumiendo que las proporciones de fumadores en cada una de las muestras sean muy próximas a las proporciones poblacionales, ver Tabla 2.

**Tabla 2.** **Descripción del muestreo Caso-Control A, donde D+ son enfermos y D^–^ sanos**.

	Fuman	No Fuman	TOTAL	
D +	160	40	200	P_1_ = 0,80
D^–^	6	19	25	P_0_ = 0,24
Total	N_1_ = 166	N_0_ = 59		
	R_1_ = 0,96	R_0_ = 0,68	RR = 0,96/0,68 = 1,41	

Vemos que sumando por columnas aparecen 166 fumadores, de los cuales son enfermos 160. El presunto R_1_ sería 160/166 = 0,96, que no se aproxima nada a 0,40 de la población. Y sumando por columnas aparecen 59 no fumadores, de los cuales son enfermos 40. El presunto R_0_ sería 40/59 = 0,68, que no se aproxima nada al 0,05 de la población. El presunto RR sería 0,96/0,68 = 1,41, que no se aproxima nada al 8 de la población.

B - Muestreo Caso-Control tomando una muestra aleatoria de 20 enfermos y otra muestra aleatoria de 2500 sanos y asumiendo que las proporciones de fumadores en cada una de las muestras sean muy próximas a las proporciones poblacionales, ver Tabla 3.

**Tabla 3.** **Descripción del muestreo Caso-Control B, donde D+ son enfermos y D^–^ sanos**.

	Fuman	No Fuman	TOTAL	
D +	16	4	20	P_1_ = 0,80
D^–^	600	1900	2500	P_0_ = 0,24
Total	N_1_ = 616	N_0_ = 1904		
	R_1_ = 0,026	R_0_ = 0,002	RR = 13,00	

Vemos que sumando por columnas aparecen 616 fumadores, de los cuales son enfermos 16. El presunto R_1_ sería 16/616 = 0,026, que no se aproxima nada a 0,40 de la población (ni al 0,96 del muestreo anterior). Y sumando por columnas aparecen 1904 no fumadores, de los cuales son enfermos 4. El presunto R_0_ sería 4/1904 = 0,002, que no se aproxima nada al 0,05 de la población. (ni al 0,68 del muestreo anterior). El presunto RR sería 0,026/0,002 = 13,00, que no se aproxima nada al 8 de la población (ni al 1,4 del muestreo anterior).

Por tanto, con muestreo Caso-Control sólo se puede estimar P_1_ y P_0_ y el cociente P_1_/P_0_, que en general es distinto de R_1_/R_0_. Pero con los estimadores de P_1_ y P_0_, calculamos la OR muestral que estima la poblacional (y que es la misma calculada con las P’s que con las R’s). Y, puesto que, con enfermedades de baja frecuencia, digamos menor del 0,10, el RR = R_1_/R_0_ es muy próximo a la OR, el muestreo Caso-Control permite estimar el OR poblacional y también el RR poblacional [2].

## 3. Ventajas e Inconvenientes de Los Diseños Caso-Control

A - En algunos casos el diseño Caso-Control permite ahorrar tiempo, trabajo y costes [5]. Por ejemplo, para ver si el consumo excesivo y prolongado de alcohol durante la juventud aumenta el riesgo de demencia senil, un estudio prospectivo debe seguir durante cuatro decenios a dos grupos de jóvenes, con y sin ese hábito, y contar el número de ellos que desarrollan demencia en la vejez. El diseño Caso-Control, toma dos muestras de ancianos actuales, uno con demencia y otro sin esa patología, pregunta a cada anciano (o sus allegados) si bebía en exceso en su juventud y calcula la proporción correspondiente. La muestra podría tomarse en pocos días.

B - En algunos casos el diseño Caso-Control tiene mucha más potencia estadística que el prospectivo. Consideremos, por ejemplo, una población en la que tenían ese habito el 10% de los jóvenes y al llegar a la vejez padecían demencia el 9 de cada mil, mientras que en los no bebedores padecían demencia senil uno cada mil. Beber multiplica por 9 el riesgo de demencia senil. Con un estudio Caso-Control en que se pregunta a 70 ancianos con demencia y a otros 70 ancianos sin demencia si habían tenido ese habito en la juventud, la potencia estadística (probabilidad de detectar esa asociación) es 97%. Es decir, 97 de cada 100 estudios daría un resultado muy significativo. Con estudio prospectivo en que se sigue a 70 jóvenes con ese hábito y otros 70 sin él, hay que esperar 40 años para ver cuantos hacen demencia en cada grupo y la potencia estadística es de 0,01%, es decir, solo uno cada diez mil estudios como ese nos daría un resultado muy significativo. Para tener la misma potencia que con el diseño Caso-Control (97%), en un estudio prospectivo habría que estudiar más de 4000 personas en cada grupo.

C - En los estudios Caso-Control suele haber muchos más factores de confusión que en los prospectivos [5]. Parte de ellos podrán neutralizarse con análisis estratificado, pero otros probablemente ni siquiera habrán sido recogidos en el estudio. En particular el sesgo de memoria puede ser muy acusado. Por lo que los resultados deben ser valorados con prudencia y siempre contemplando la posibilidad de confirmarlos con estudios prospectivos. Pero si ello no es posible y la asociación encontrada es altamente significativa, los resultados pueden ser considerados como indicio importante.

## 4. Conclusión

Con muestreo Caso-Control no se pueden estimar R_1_ ni R_0_ ni RR, porque los valores muestrales que estimarían R_1_ y R_0_ dependen de la cantidad de Casos y Controles que se haya decidido tomar. El interés del investigador es estimar el RR poblacional, para lo cual lo más lógico es hacer un muestreo prospectivo. Sin embargo, en muchas ocasiones el muestreo caso-control es más barato, a veces el único factible y en otras ocasiones incluso más potente que el prospectivo. Además, cuando la enfermedad es poco prevalente, y son R_1_ y R_0_ pequeños, la OR de la muestra Caso-Control estima con buena aproximación el RR poblacional. Por tanto, los estudios de tipo caso-control, si bien presentan limitaciones de sesgo, no deben ser subestimados. Sus resultados deben ser valorados con prudencia y siempre contemplando la posibilidad de confirmarlos con estudios prospectivos. Pero si ello no es posible y la asociación encontrada es altamente significativa, los resultados se pueden considerar como indicio importante.

## Data Availability

Los datos contenidos en el manuscrito son ficticios con finalidad docente.

## Contribuciones de los Autores

CC aporto las ideas principales del texto y se encargó de su redacción. LP colaboró aportando algunos ejemplos aclaratorios y realizando la búsqueda de bibliografía complementaria. Dos autores han contribuido en la preparación del manuscrito y en los cambios editoriales. Dos autores han leído y aprobado la versión final del manuscrito. Dos autores han participado plenamente en este trabajo y están de acuerdo en asumir la responsabilidad de todos los aspectos de este trabajo.

## Aprobación Ética y Consentimiento Informado

No aplicable.

## Agradecimientos

No aplicable.

## Financiación

Esta investigación no recibió financiación externa.
